# Alcohol affordability: implications for alcohol price policies. A cross-sectional analysis in middle and older adults from UK Biobank

**DOI:** 10.1093/pubmed/fdab095

**Published:** 2021-04-09

**Authors:** Simon C Moore, Bella Orpen, Jesse Smith, Chinmoy Sarkar, Chenlu Li, Jonathan Shepherd, Sarah Bauermeister

**Affiliations:** Violence Research Group, School of Dentistry, Cardiff CF14 4XY, UK; Crime and Security Research Institute, Cardiff University, Cardiff CF10 3AE, UK; Crime and Security Research Institute, Cardiff University, Cardiff CF10 3AE, UK; Centre for the Development and Evaluation of Complex Interventions for Public Health Improvement, School of Social Sciences, Cardiff University, Cardiff CF10 3BD, UK; Healthy High Density Cities Lab, HKUrbanLab, The University of Hong Kong, Hong Kong; Deloitte LLP, London EC4A 3HQ, UK; Violence Research Group, School of Dentistry, Cardiff CF14 4XY, UK; Crime and Security Research Institute, Cardiff University, Cardiff CF10 3AE, UK; Department of Psychiatry, University of Oxford, Warneford Hospital, Oxford OX3 7JX, UK

**Keywords:** alcohol consumption, public health

## Abstract

**Background:**

Increasing the price of alcohol reduces alcohol consumption and harm. The role of food complementarity, transaction costs and inflation on alcohol demand are determined and discussed in relation to alcohol price policies.

**Methods:**

UK Biobank (*N* = 502,628) was linked by region to retail price quotes for the years 2007 to 2010. The log residual food and alcohol prices, and alcohol availability were regressed onto log daily alcohol consumption. Model standard errors were adjusted for clustering by region.

**Results:**

Associations with alcohol consumption were found for alcohol price (*β* = −0.56, 95% CI, −0.92 to −0.20) and availability (*β* = 0.06, 95% CI, 0.04 to 0.07). Introducing, food price reduced the alcohol price consumption association (*β* = −0.26, 95% CI, −0.50 to −0.03). Alcohol (*B* = 0.001, 95% CI, 0.0004 to 0.001) and food (*B* = 0.001, 95% CI, 0.0005 to 0.0006) price increased with time and were associated (*ρ* = 0.57, *P* < 0.001).

**Conclusion:**

Alcohol and food are complements, and the price elasticity of alcohol reduces when the effect of food price is accounted for. Transaction costs did not affect the alcohol price consumption relationship. Fixed alcohol price policies are susceptible to inflation.

## Introduction

Fluctuations in the price of goods correspond with a change in the demand for those goods, otherwise known as the price elasticity of demand (PED).^1^ This applies to alcohol.^2–4^ An increase in alcohol retail price corresponds with a reduction in alcohol consumption and alcohol-related health harms.^5–8^ The evidence for this relationship between alcohol retail price and the demand for alcohol is mostly derived from studies examining variations in alcohol retail price over time.^4^^,^^9^ There are also several natural experiments in, for example, Finland and Canada^3^^,^^6^ where an abrupt change in alcohol price coincided with a change in alcohol-related health harms. This evidence has been used to motivate the implementation of a minimum unit (8 g ethanol) price (MUP) to challenge alcohol-related harm in Scotland^10^ and Wales^11^ in the UK. The estimates used to inform these policies broadly suggest a PED of ~ −0.5 for alcohol,^4^^,^^12^ meaning that for every 1% increase in alcohol price 0.5 fewer units of alcohol are consumed. These PED estimates informed a MUP of £0.50 in Scotland (May 2018) and Wales (March 2020).

While increasing alcohol retail price is regarded as one of the more effective policies available to reduce the harms from alcohol,^13^ price is only a component of the demand for alcohol. Both monetary and non-monetary factors influence consumer’s perception of retail price,^14–16^ meaning that it is alcohol affordability, the meaning given to price by consumers, that is the more appropriate operationalization of price. Affordability is typically defined as a function of price and household income, or residual income following the decision to purchase.^17–20^ This is notable as other costs should be expected to therefore influence residual income and therefore the demand for alcohol. Here we seek to develop the alcohol affordability construct and consider factors that are expected to influence the demand for alcohol and any implications for alcohol price policy.

First, in Canada and Finland the governments hold a monopoly over alcohol retail^3^^,^^6^: alcohol is typically sold in establishments separated from grocery stores. Conversely, alcohol is not subject to a state monopoly in the UK; it is available through various retail outlets, the prominent suppliers of which are local convenience stores and large grocery stores. In these retail outlets consumers can purchase alcohol, and other household goods alongside food.^21^^,^^22^ Consumers typically budget for their consumption^23^^,^^24^ and as food, a necessity, and alcohol, a luxury, are typically sold with a single point of sale in the UK, it is plausible that the price of food might influence the demand for alcohol. There are two ways to define such a relationship. First, that alcohol is a substitute to food, in which case the demand for alcohol would increase as the price of food increases (consumers spend less on food and purchase more alcohol in its place), or second, as a complement, when the price of food increases the demand for alcohol decreases. This relationship is formalized in Equation (1).

(1) \begin{equation*} \frac{\left(\Delta{Q}_A/{Q}_A\right)}{\left(\Delta{P}_F/{P}_F\right)} \end{equation*}

Q_A_ is the quantity of alcohol consumed and P_F_ is the price of food. If Equation 1 is negative alcohol is a complement to food, if it is positive it is a substitute.^25^ The implication is that a complementary relationship would imply that food price could be a component of the demand for alcohol and this has not been appropriately determined.^4^^,^^9^

Second, both an increase in alcohol retail price and an increase in alcohol availability are associated with alcohol-related harm.^13^^,^^26–28^ In Canada it is observed that retail price and availability are independent, in respect of the demand for alcohol, and it is reasoned that greater availability means longer opening hours, and greater competition between outlets.^26^ We suggest a more parsimonious account placing availability as a feature of alcohol affordability. Greater availability implies easier access to alcohol and therefore lower transaction costs, the costs involved with transferring goods in store for consumption at home,^29^ due to, for example, lower travel costs. If alcohol availability and alcohol retail price are associated, as might be expected with greater competition where density is greater, then this may affect estimates of the PED for alcohol.

Finally, household income, a determinant of alcohol affordability, typically increases over time^30^ and does so variously by geographic region^31^ and industry sector.^32^ There is also a corresponding increase in retail price overtime.^33^ For example, the UK Consumer Price Index increased from 72.7 in 2000 to 108.8 in 2020.^34^ This is notable as the implementation of MUP^10^^,^^11^ is not index linked. If the price of beverages susceptible to MUP does not increase with inflation, then the affordability of these beverages is expected to increase over time, as a percentage of residual household income. The utility of a fixed MUP, and therefore estimates of the harms saved, should be expected to wane.

We assess the implications of complementarity, transaction costs and inflation on the demand for alcohol using UK Biobank cohort data linked to retail price data. In addition, models control for a range of individual and household characteristics variously associated with preferences for alcohol.

## Methods

Between 2006 and 2010, a population sample of 502 649 adults aged 40–73 years participated in the UK Biobank prospective cohort study at 22 research centres (Appendix 1) located across the UK.^35^ Participants were registered with the UK National Health Service (NHS) and lived within a radius of 40 km from one of the research centres. Self-reported data were collected using touch-screen questionnaires and interviews^35^ and participant responses were record linked to routine NHS health data. Information on the assessment procedure, protocol and information on data access is available online (www.ukbiobank.ac.uk).

### Data

#### Biobank

Ethical approval was granted to UK Biobank from the NHS Health Research Authority, Research Ethics Committee (reference 11/NW/0382). The current study was conducted using the UK Biobank Resource and pre-registered (approved application number 15008).

Biobank participants (mean age 57.03 years, SD = 8.09) were sampled from March 2006 to October 2010; 40,658 were non-drinkers (either former drinker or never drinker) and excluded. Drinkers reported either their average weekly or average monthly glasses of alcoholic beverages consumed (red wine, champagne or white wine, pint of beer or cider, spirits, fortified wine, other). Beverages were converted to grams of ethanol per typical glass (12 g red wine, 11.3 g white wine, 19.9 g beer, 9.2 g spirits, 9.6 g fortified wine). The question relating to ‘other’ alcoholic beverage specified alcopops containing an estimated 12 g alcohol. There were 387 160 available responses with consumption data (mean = 20.07 g/day, min = 0 g/day, max = 904.97 g/day) and following case-wise deletion on other regression variables, the final analytic sample was 226 548 participants (mean = 20.75 g/day, SD = 19.78).

#### Price data

The use of unweighted Office for National Statistics (ONS) price data follows methods described elsewhere.^36^ The ONS samples typically consumed services and items to derive the UK Retail Price and Consumer Price indices. The methodology describing the sampling is available elsewhere.^37^ The prices are collected by region (Appendix 1) each month. They are described by high level Divisions (Appendix 2), and more granular Groups and Classes. Two Divisions are of interest here, ‘Food and Non-Alcoholic Beverages’ and ‘Alcoholic Beverages and Tobacco’, these include the Groups ‘Alcoholic beverages’, ‘Tobacco’ and ‘Non-alcoholic beverages’ allowing prices for alcohol, tobacco and food to be separated. The unweighted average food item price was calculated by month and year and merged into the UK Biobank data by region (Appendix 1), and the month and year when participants completed the survey, as was the average price of alcohol.^36^ As the primary hypothesis concerns the retail sale of alcohol, alcohol was further described on an item by item basis according to whether the price quote was for on-trade (e.g. bars, nightclubs) or off-trade (e.g. grocery stores) by description (e.g. ‘bottle of lager in nightclub’) and price.^36^ The on-trade sale of alcohol carries a price premium therefore allowing items such as ‘spirit-based drink 275 ml’ to be identified as an on-trade beverage (Appendix 3). On-trade items were dropped. Finally, price quotes for several items in 2006 were missing descriptions and were apparently unique. Given 0.76% of UK Biobank participants were recruited in 2006, these data were dropped.

#### Alcohol availability

The UK Biobank Urban Morphometric Platform^38^ contains data concerning the neighbourhood in which residents live, including details of the density of premises licensed for the sale of alcohol within multiscale catchments of each UK Biobank participants’ residence. The variables describing the density of licensed premises (Public House, Bar or Nightclub; Restaurant or Cafeteria; Other Licensed Premise) within 1000 m were associated (Spearman *ρ* > 0.42, *P* < 0.001, for each comparison) and were reduced to a single ‘availability’ index using factor analysis (factor loadings were, respectively, 0.805, 0.821, 0.582).

#### Covariates

ICD10 diagnostic codes were available for all primary, secondary and external causes across all hospital inpatient records in UK Biobank. These were searched for alcohol-specific codes (Appendix 4)^39^ across all coding fields.^40^ Those with one or more alcohol-codes were identified and this binary variable included in analyses. Access to a car was included. Large out of town grocery stores have used alcohol as a loss-leader to attract custom^41^ and access to a car may therefore effect transaction costs. Additional demographic, and socioeconomic indicators were further included (Table 1). Missing values on household income were imputed using the median category (£31 000 to £51 999).

**Table 1:** Descriptive statistics

	*Mean*	*Proportion*	95% *CI*	
			*Lower*	*Upper*
Alcohol consumption (g/day)	20.751		20.669	20.832
Alcohol price (£)	9.917		9.916	9.919
Food price (£)	2.470		2.469	2.470
Alcohol availability				
Public house/bar/night club	2.696		2.678	2.714
Restaurant/zafeteria	4.123		4.087	4.159
Other licensed premise/vendor	0.925		0.919	0.932
Household income				
< £18 000		0.160	0.159	0.162
£18 000 to £30 999		0.223	0.221	0.224
£31 000 to £51 999		0.355	0.353	0.357
£52 000 to £100 000		0.205	0.204	0.207
> £100 000		0.056	0.056	0.057
Socioeconomic status (Townsend)	−1.490		−1.502	−1.479
General health				
Excellent		0.174	0.172	0.176
Good		0.601	0.599	0.603
Fair		0.192	0.191	0.194
Poor		0.032	0.032	0.033
Age (years)	56.848		46.815	56.880
Gender (male = 1)		0.498	0.496	0.500
Ethnicity (white = 1)		0.034	0.033	0.034
Qualifications				
College or university		0.357	0.355	0.359
None		0.132	0.131	0.134
Employment				
Paid employment		0.603	0.601	0.605
Retired		0.325	0.323	0.327
Sick		0.021	0.021	0.022
Unemployed		0.015	0.014	0.015
Voluntary		0.032	0.032	0.033
Student		0.002	0.002	0.002
Uses tobacco		0.069	0.068	0.070
Access to a car		0.837	0.832	0.842
Activity				
Moderate (days/week)		3.622	3.612	3.632
Vigorous (days/week)		1.912	1.904	1.920
Walk (days/week)		5.411	5.403	5.419
Reducing alcohol consumption				
For health reasons		0.175	0.174	0.177
For other reasons		0.213	0.212	0.215
Self-diagnosis				
Neurological condition		0.019	0.019	0.020
Alcohol problem		0.001	0.000	0.001
Alcohol-specific ICD10 code		0.004	0.003	0.004
Number of household occupants				
1		0.168	0.167	0.170
2		0.473	0.471	0.476
3		0.159	0.157	0.160
4		0.145	0.144	0.147
5		0.040	0.039	0.041
6		0.008	0.008	0.008
>6		0.006	0.006	0.007
Home owed outright		0.528	0.526	0.530
Home owned with mortgage		0.392	0.390	0.394
Season				
Spring		0.279	0.277	0.281
Summer		0.262	0.260	0.263
Autumn		0.261	0.259	0.262
Winter		0.199	0.197	0.201

#### Analysis

Only respondents with non-zero alcohol consumption were included. Data were analysed using Ordinary Least Square regression in Stata MP v16.0,^42^ with standard errors corrected using ONS region.

## Results

**Fig. 1:**
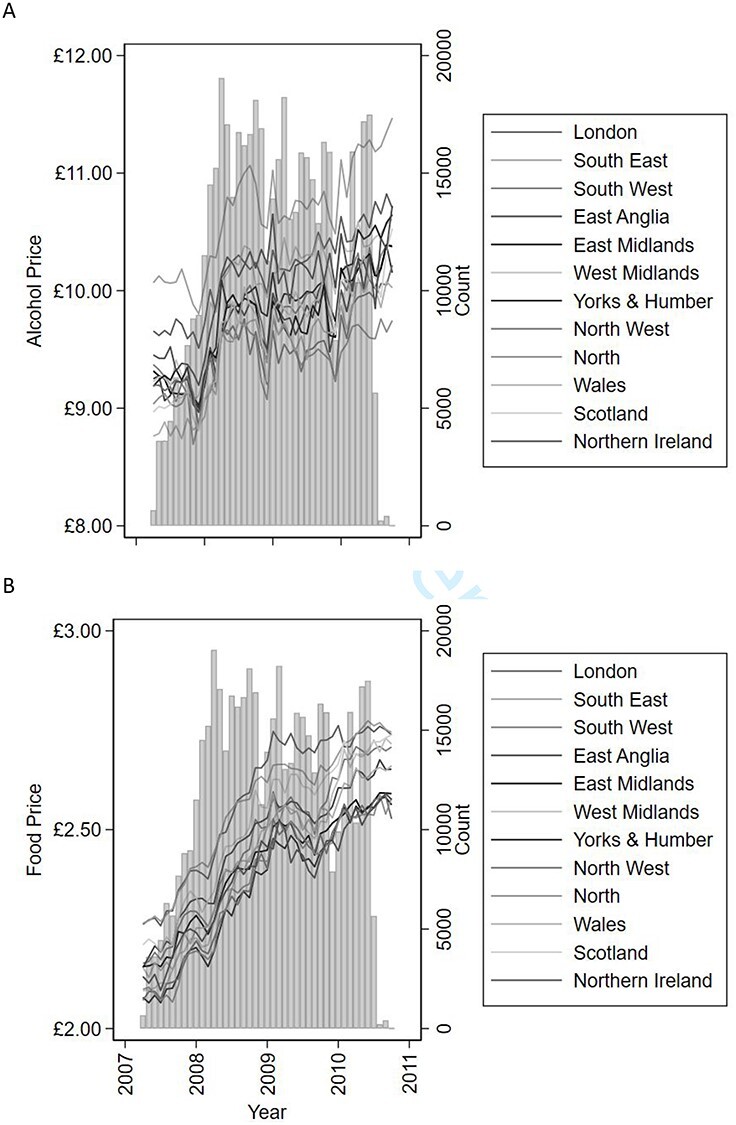
Number of UK Biobank participants recruited by month (panel **A** and panel **B**, vertical columns), overlaid with the average monthly price of alcohol across the 12 regions (panel A) and the average monthly price of food across the 12 regions (panel B).

The ONS average price of alcohol was £9.83 (SD = 0.52, min = £8.69, max = £11.47) and the average price of food was £2.44 (SD = 0.18, min = £2.06, max = £2.77; Appendix 5). Both alcohol (B = 0.001, 95% CI 0.0004 to 0.001) and food (B = 0.001, 95% CI = 0.0005 to 0.0006) price increased linearly with time (Fig. 1), and an ordered logit also yielded a positive relationship between year of survey and household income (*B* = 0.088, 95% CI, 0.080 to 0.097).

While the planned analyses treated the available data as cross-sectional, the temporal aspect of the price data both had the advantage of variation across time and region, but required the data were detrended before inclusion in models. The predicted residuals had the minimum price added, plus one, so that the range did not cross zero, and the log was taken. Unadjusted regression (*n* = 226,608 for all analyses) of standardized log alcohol price on log alcohol consumption yielded a significant relationship (*b* = −0.64, 95% CI –0.66 to −0.61) and between the log price of food and log alcohol consumption (*b* = −1.48, 95% CI = −1.52 to −1.44). Table 1 presents descriptive statistics and Table 2 the full regression results. Log residual alcohol price and log residual food price were correlated (*ρ* = 0.57, *P* < 0.001), however the variance inflation factors (VIFs) suggest this association was not of concern (alcohol VIF = 1.60, food VIF = 1.63).

As food price and alcohol availability were added (Table 2) the coefficient on alcohol price reduced from −0.56 to −0.26 (Chow test *χ*^2^ = 13.76, *P* < 0.001). The coefficient on food price was negative (Model 4, Table 2), implying that the quantity of alcohol consumed decreases as the price of food increases.

The fully adjusted model (Model 4, Table 2) was repeated, stratifying by level of alcohol consumption. The current UK guidelines are that individuals should not consume more than 14 units (where 1 unit is 8 g ethanol) each week, equivalent to 16 g ethanol/day. Coefficients were compared for low consumption (<= 16 g/day, *n* = 119,461) to coefficients for those drinking over 16 g/day (*n* = 101 960). For each of alcohol price (low consumption *β* = −0.35, 95% CI, −0.38 to −0.32; high consumption *β* = 0.005, 95% CI, −0.01 to 0.02; *χ*^2^ = 5.31, *P* = 0.02), food price (low consumption *β* = −1.00, 95% CI = −1.06 to −0.94; high consumption *β* = −0.13, 95% CI, −0.16 to −0.09; *χ*^2^ = 8.26, *P* < 0.01) and availability (low consumption *β* = 0.05, 95% CI, 0.05 to 0.06; high consumption *β* = 0.01, 95% CI, 0.003 to 0.01; *χ*^2^ = 79.23, *P* < 0.001) coefficients significantly fell moving from low to high alcohol consumers, although there is no significant effect of alcohol price on alcohol consumption for high consumption individuals. Thus alcohol demand is more inelastic to changes in price for high alcohol consumers.

Finally, the fully adjusted model (Model 4, Table 2) was repeated stratifying by SES quintile to explore the policy relevance of the results by deprivation (Table 3).

Consistent with expectations, as the availability of alcohol increased, so did consumption. Household income and number of occupants, both likely to influence household finances, were associated with alcohol consumption. Those who had contacted healthcare services for alcohol-related reasons and those who self-diagnosed as having a ‘problem’ with alcohol consumed more alcohol, whereas those in general poor health consumed less alcohol. Students consumed less alcohol than their non-student peers, although given the age (mean age = 48.33 years, SD = 6.37) of the cohort UK Biobank students might be more mature than typically aged UK students.

## Discussion

### Main finding of this study

The initial relationship between price and alcohol consumption suggested a price elasticity of −0.56, within the margin of error of previous estimates.^4^^,^^12^ However, including the price of food, which is associated with the price of alcohol over time, reduced this estimate to −0.26, half previous estimates. This complementary relationship deserves further attention. In particular whether this is unique to countries where alcohol is sold alongside food^21^^,^^22^ and whether it generalizes to jurisdictions where alcohol is retailed separately from usual groceries. If the way alcohol is retailed influences consumption then consideration should be given to how alcohol is retailed in the UK and whether separate stores for alcohol and food, as is found in Canada and Finland for example, provide greater opportunity to challenge the harms associated with alcohol consumption. Furthermore, if the complementary relationship between alcohol and food described here is a consequence of how alcohol is retailed then pricing policies might be undermined if retailers offset an increase in alcohol price by reducing the price of food or increase the availability of alcohol.

Consistent with similar work, there was a robust dose–response relationship between income and alcohol consumption,^19^^,^^43^ confirming the role of affordability, which we broadened to include alcohol availability. Greater availability reduces transaction costs, a construct that may explain the relationship between access to a car and alcohol consumption as a car provides easier access to less expensive out-of-town grocery stores.^41^^,^^44^ We suggest that consumers are versatile, willing to alter how they acquire alcohol to maximize affordability, and may make greater use of local convenience stores as alcohol price increases as MUP is introduced^44^ as doing so reduces transaction costs. However, how different consumer groups might budget for alcohol alongside necessities such as food, and whether food is a necessity for those who are alcohol dependant deserves attention. Health, diet and alcohol are related,^45^^,^^46^ notably for dependant drinkers where a thiamine hydrochloride nutritional deficiency can contribute to brain injury.^47^^,^^48^ The effect of alcohol availability on consumption, however, did not influence the relationship between price and consumption, consistent with previous findings.^26^

In the UK, MUP has been set at £0.50 in Scotland^10^ and Wales^11^ but has not been index linked, meaning that the £0.50 MUP remains constant over time. Price inflation and an increase in household income was notable, stable characteristics of the UK economy. MUP imposes greater profit margins on low-cost alcohol and it is feasible that the retail price of these items will not increase inline with inflation. Residual household income for items susceptible to MUP may decline and the affordability of these items will increase. It would be perspicacious to adjust MUP according to the retail price and income indices. Without doing so the anticipated effectiveness of MUP should be expected to wane following implementation.

**Table 2:** Full regression models for the price of alcohol, the price of food and alcohol availability on alcohol consumption

*Alcohol consumption (log g/day)*	*Model 1*				*Model 2*				*Model 3*				*Model 4*			
	*β*	95% CI		*P*	*β*	95% CI		*P*	*β*	95% CI		*P*	*β*	95% CI		*P*
		Lower	Upper			Lower	Upper			Lower	Upper			Lower	Upper	
Log alcohol price	−0.559	−0.924	−0.195	0.013	−0.264	−0.500	−0.028	0.036	−0.569	−0.944	−0.194	0.014	−0.261	−0.498	−0.025	0.037
Log food price					−1.020	−1.399	−0.641	0.002					−1.066	−1.446	−0.686	0.001
Alcohol availability									0.049	0.028	0.071	0.003	0.056	0.038	0.074	0.001
Household income (reference: <£18 000)																
£18 000 to £30 999	0.073	0.057	0.089	<0.001	0.082	0.059	0.105	0.001	0.071	0.056	0.085	<0.001	0.080	0.059	0.101	<0.001
£31 000 to £51 999	0.108	0.061	0.156	0.003	0.125	0.065	0.184	0.004	0.105	0.062	0.147	0.002	0.121	0.066	0.176	0.004
£52 000 to £100 000	0.220	0.128	0.312	0.003	0.247	0.141	0.354	0.003	0.213	0.129	0.298	0.002	0.241	0.141	0.340	0.003
> £100 000	0.348	0.141	0.554	0.009	0.391	0.172	0.610	0.008	0.333	0.142	0.525	0.008	0.376	0.173	0.579	0.007
Socioeconomic status (Townsend)	0.020	0.007	0.033	0.013	0.024	0.006	0.042	0.020	0.015	0.002	0.028	0.034	0.018	0.001	0.036	0.045
General health (reference: excellent)																
Good	−0.004	−0.021	0.013	0.540	−0.006	−0.022	0.011	0.390	−0.003	−0.020	0.014	0.638	−0.005	−0.021	0.012	0.477
Fair	−0.029	−0.075	0.017	0.151	−0.031	−0.079	0.016	0.137	−0.027	−0.072	0.018	0.167	−0.029	−0.075	0.017	0.152
Poor	−0.131	−0.187	−0.075	0.003	−0.132	−0.187	−0.078	0.003	−0.128	−0.182	−0.075	0.003	−0.129	−0.181	−0.078	0.002
Age (months/100)	−0.062	−0.103	−0.021	0.014	−0.054	−0.094	−0.014	0.020	−0.062	−0.102	−0.022	0.013	−0.054	−0.093	−0.014	0.019
Gender (male = 1)	0.758	0.679	0.837	<0.001	0.754	0.678	0.830	<0.001	0.757	0.678	0.835	<0.001	0.753	0.677	0.829	<0.001
Ethnicity (white = 1)	−0.685	−0.978	−0.392	0.003	−0.642	−0.956	−0.327	0.005	−0.682	−0.965	−0.400	0.003	−0.637	−0.937	−0.336	0.004
Qualifications																
College or university	−0.053	−0.111	0.005	0.066	−0.046	−0.107	0.015	0.106	−0.058	−0.117	0.001	0.052	−0.052	−0.114	0.011	0.084
None	0.036	−0.021	0.094	0.154	0.023	−0.035	0.080	0.334	0.039	−0.016	0.094	0.122	0.025	−0.030	0.081	0.278
Employment (reference: paid employment)																
Retired	0.034	0.020	0.049	0.003	0.031	0.018	0.043	0.003	0.036	0.021	0.050	0.003	0.032	0.019	0.044	0.002
Sick	0.070	0.010	0.130	0.031	0.064	−0.003	0.131	0.058	0.073	0.015	0.131	0.025	0.066	0.001	0.132	0.049
Unemployed	0.078	0.027	0.130	0.014	0.094	0.033	0.155	0.013	0.079	0.027	0.131	0.014	0.095	0.034	0.156	0.012
Voluntary	−0.015	−0.053	0.023	0.340	−0.007	−0.041	0.027	0.577	−0.016	−0.053	0.020	0.286	−0.009	−0.040	0.023	0.494
Student	−0.065	−0.123	−0.007	0.036	−0.059	−0.103	−0.015	0.021	−0.066	−0.125	−0.008	0.035	−0.060	−0.104	−0.016	0.019
Uses tobacco	0.370	0.294	0.446	<0.001	0.371	0.291	0.450	<0.001	0.370	0.291	0.448	<0.001	0.370	0.288	0.452	<0.001
Access to a car	0.009	0.005	0.013	0.003	0.006	0.001	0.011	0.025	0.011	0.007	0.015	0.001	0.008	0.004	0.012	0.007
Activity																
Moderate (days/week)	<0.001	−0.003	0.003	0.873	0.001	−0.002	0.003	0.389	<0.001	−0.002	0.003	0.759	0.001	−0.001	0.003	0.273
Vigorous (days/week)	0.007	0.001	0.013	0.033	0.007	0.001	0.013	0.035	0.007	0.001	0.013	0.029	0.007	0.001	0.013	0.029
Walk (days/week)	0.015	0.008	0.023	0.005	0.015	0.008	0.023	0.005	0.014	0.007	0.021	0.005	0.014	0.007	0.022	0.006
Reducing alcohol consumption																
For health reasons	−0.193	−0.212	−0.174	<0.001	−0.193	−0.209	−0.177	<0.001	−0.194	−0.213	−0.175	<0.001	−0.193	−0.209	−0.178	<0.001
For other reasons	−0.489	−0.542	−0.436	<0.001	−0.488	−0.543	−0.433	<0.001	−0.488	−0.540	−0.436	<0.001	−0.487	−0.542	−0.432	<0.001
Self-diagnosis																
Neurological condition	−0.040	−0.078	−0.003	0.041	−0.043	−0.084	−0.002	0.043	−0.040	−0.077	−0.003	0.041	−0.043	−0.084	−0.002	0.043
Alcohol problem	0.652	0.501	0.804	<0.001	0.650	0.474	0.827	0.001	0.651	0.499	0.804	<0.001	0.649	0.471	0.827	0.001
Alcohol-specific ICD10 code	0.580	0.388	0.773	0.001	0.582	0.398	0.766	0.001	0.579	0.384	0.775	0.001	0.581	0.393	0.769	0.001
Number of household occupants (reference = 1)																
2	−0.009	−0.035	0.018	0.414	−0.018	−0.036	<0.001	0.048	−0.004	−0.032	0.024	0.725	−0.013	−0.032	0.006	0.127
3	−0.087	−0.099	−0.075	<0.001	−0.093	−0.109	−0.076	<0.001	−0.080	−0.095	−0.065	<0.001	−0.085	−0.103	−0.067	<0.001
4	−0.136	−0.153	−0.118	<0.001	−0.138	−0.157	−0.119	<0.001	−0.128	−0.149	−0.107	<0.001	−0.130	−0.150	−0.109	<0.001
5	−0.183	−0.227	−0.138	<0.001	−0.180	−0.217	−0.144	<0.001	−0.175	−0.226	−0.124	0.001	−0.172	−0.215	−0.128	<0.001
6	−0.194	−0.292	−0.095	0.005	−0.189	−0.293	−0.086	0.007	−0.185	−0.278	−0.093	0.005	−0.180	−0.275	−0.084	0.006
>6	−0.179	−0.306	−0.052	0.017	−0.175	−0.307	−0.044	0.021	−0.175	−0.297	−0.053	0.017	−0.171	−0.297	−0.044	0.020
Home owed outright	0.069	0.033	0.105	0.006	0.065	0.029	0.102	0.008	0.069	0.026	0.111	0.011	0.065	0.021	0.109	0.015
Home owned with mortgage	0.096	0.059	0.134	0.002	0.090	0.054	0.127	0.002	0.097	0.052	0.141	0.004	0.091	0.046	0.135	0.005
Season (reference: spring)																
Summer	−0.043	−0.088	0.002	0.057	−0.044	−0.096	0.008	0.079	−0.045	−0.086	−0.005	0.036	−0.047	−0.093	−0.001	0.047
Autumn	−0.098	−0.166	−0.030	0.016	−0.087	−0.166	−0.007	0.039	−0.099	−0.165	−0.033	0.014	−0.088	−0.166	−0.009	0.036
Winter	−0.066	−0.125	−0.007	0.035	−0.024	−0.077	0.029	0.281	−0.067	−0.125	−0.010	0.031	−0.023	−0.075	0.029	0.279
Constant	2.977	2.678	3.277	<0.001	2.938	2.592	3.284	<0.001	2.980	2.680	3.280	<0.001	2.939	2.591	3.287	<0.001

**Table 3:** Fully adjusted coefficients for alcohol price and log food price on log alcohol consumption (g/day) by socioeconomic quintile (a greater value represents greater deprivation)

		*Alcohol (g/day)*	*Log alcohol price*	*Log food price*
*SES Townsend*	*n*	*Mean* (95% CI)	β (95% CI)	β (95% CI)
−6.26 to −3.95	45 328	19.753 (19.589 to 19.917)	−0.159 (−0.350 to 0.031)	−1.187^***^ (−1.624 to −0.751)
−3.96 to −2.83	45 343	19.898 (19.728 to 20.068)	−0.217^**^ (−0.377 to −0.057)	−1.101^***^ (−1.513 to −0.689)
−2.84 to −1.48	45 294	20.196 (20.020 to 20.373)	−0.217^**^ (−0.361 to −0.072)	−1.238^***^ (−1.568 to −0.908)
−1.49 to 0.93	45 322	21.114 (20.923 to 21.305)	−0.270^**^ (−0.435 to −0.105)	−1.100^***^ (−1.386 to −0.815)
0.94 to 10.16	45 321	23.118 (22.894 to 23.343)	−0.486^*^ (−0.859 to −0.112)	−0.747^*^ (−1.334 to −0.161)

^***^ *P* < 0.001,

^**^ *P* < 0.01,

^*^ *P* < 0.05.

While the emphasis here is on financial disincentives to consume alcohol, it is notable that SES was associated with consumption. It is established that alcohol-related harm is greater in more deprived communities, despite the belief that there are comparable or lower levels of reported alcohol consumption compared to less deprived groups.^49–51^ The current analyses challenge this latter assumption, finding that alcohol consumption increases as deprivation increases. Moreover, the PED for alcohol increases as deprivation increases, suggesting that price policies maybe more effective in more deprived communities where both consumption and harm is greatest.

### Limitations

The results presented here must be interpreted according to the limitations involved with cross-sectional research. Causation cannot be inferred, and it is feasible that those who choose to drink more alcohol are drawn to areas where alcohol and food are cheaper. This is partially offset by arguments that the demand for alcohol is causally associated with alcohol retail price.^52^ Further, UK Biobank is specific to those in middle and later life. However, in the UK, the age group most likely to experience alcohol-related hospital admission are between 45 and 64 years of age and 57% of all alcohol-specific deaths occur in the 50- to 69-year age group,^53^ suggesting the cohort considered here is relevant. Finally, there may be fixed regional differences in alcohol consumption that may confound estimates and that cannot be accounted for in the cross-sectional nature of the data analysed here.

In sum, alcohol is a complement to food, a relationship that has not been acknowledged in estimates of the PED for alcohol. If the results generalize outside of the cohort considered here, then the PEDs used in modelling policy effectiveness are likely overstated. But it is also feasible that the way alcohol is retailed may influence this relationship and that therefore separating alcohol and grocery sales, as is typical in many jurisdictions, should be considered. Alcohol price policy should also recognize heterogenous alcohol consumers and that high and low consumers may be impacted by price changes in food and alcohol to different extents. Further, any alcohol price policy should be index linked to account for variations in alcohol affordability over time.

### What is already known on this subject?

As alcohol becomes more affordable alcohol consumption increases and this relationship has informed price polices, introduced to challenge alcohol-related harm.

### What this study adds?

Alcohol consumption increases as food price decreases suggesting the price of food may also influence alcohol purchase decisions. This relationship has not been accounted for in estimates of reduced alcohol-related harm due to alcohol MUP policies in the UK and it is feasible such policies are less effective than currently assumed. Alcohol transactions costs, the costs of transferring alcohol in store for consumption at home, are independent in respective of alcohol demand and have no bearing on price policy. Both food and alcohol prices are susceptible to inflation, a feature not accounted for in fixed alcohol price policies.

## Supplementary Material

20200228_Alcohol_Food_supplement_fdab095

## Notes

**Simon C. Moore**, Professor of Public Health Research

**Orpen Bella,** Research Assistant

**Smith Jesse,** Fulbright Scholar

**Sarkar Chinmoy,** Associate Professor of GIS, Urban Health and Environment

**Chenlu Li,** Risk Advisory Manager

**Jonathan Shepherd**, Emeritus Professor in Oral & Maxillofacial Surgery

**Sarah Bauermeister,** Senior Data & Science Manager
