# Supplementary material for: Alcohol affordability: implications for alcohol price policies. A cross-sectional analysis in middle and older adults from UK Biobank
Source: J Public Health (Oxf). 2021 Apr 9;44(2):e192–202. doi: 10.1093/pubmed/fdab095 (PMC9234508; doi:10.1093/pubmed/fdab095)
Supplement: 20200228_Alcohol_Food_supplement_fdab095 [file 20200228_alcohol_food_supplement_fdab095.docx]

**APPENDICES**

**Alcohol Affordability: implications for Alcohol Price Policies. A Cross-Sectional Analysis in Middle and Older Adults from UK Biobank**

**APPENDIX 1**

Translation table for ONS Region and UK Biobank Assessment Centre

| Assessment Centre | ONS Region |
| --- | --- |
| Barts, London | London |
| Birmingham | West Midlands |
| Bristol | SW |
| Bury | NW |
| Cardiff | Wales |
| Cheadle (revisit) | NW |
| Croydon | London |
| Edinburgh | Scotland |
| Glasgow | Scotland |
| Hounslow | London |
| Leeds | Yorks & Humber |
| Liverpool | NW |
| Manchester | NW |
| Middlesbrough | Yorks & Humber |
| Newcastle | Yorks & Humber |
| Nottingham | East Midlands |
| Oxford | SE |
| Reading | SE |
| Sheffield | Yorks & Humber |
| Stockport | NW |
| Stoke | West Midlands |
| Swansea | Wales |

There are three additional assessment centres in UK Biobank (Wrexham, Cheadle and Newcastle) but participants assessed at these were for either follow-up study or imaging studies only, these participants were therefore not included in the current study.

**APPENDIX 2**

ONS structure of price quotes

| DIVISION |
| --- |
| 01 Food and Non-Alcoholic Beverages |
| 02 Alcoholic Beverages and Tobacco |
| 03 Clothing and Footwear |
| 04 Housing, Water, Electricity, Gas and other Fuels |
| 05 Furniture, Household Equipment and Maintenance |
| 06 Health |
| 07 Transport |
| 08 Communications |
| 09 Recreation and Culture |
| 10 Education |
| 11 Restaurants and Hotels |
| 12 Miscellaneous Goods and Services |

| GROUP |
| --- |
| 01.1 Food |
| 01.2 Non-alcoholic beverages |
| 02.1 Alcoholic beverages |
| 02.2 Tobacco |
| 03.1 Clothing |
| 03.2 Footwear including repairs |
| 04.1 Actual rentals for housing |
| 04.3 Regular maintenance and repair of the dwelling |
| 04.4 Water Supply and misc. services for the dwelling |
| 04.5 Electricity, gas and other fuels |
| 05.1 Furniture, furnishings and carpets |
| 05.2 Household textiles |
| 05.3 Household appliances, fitting and repairs |
| 05.4 Glassware, tableware and household utensils |
| 05.5 Tools and equipment for house and garden |
| 05.6 Goods and services for routine maintenance |
| 06.1 Medical products, appliances and equipment |
| 06.2 Out-patient services |
| 06.3 In-patient service |
| 07.1 Purchase of vehicles |
| 07.2 Operation of personal transport equipment |
| 07.3 Transport services |
| 08.1 Postal services |
| 08.2 Telephone and telefax goods and services |
| 09.1 Audio-visual, photographic and information processing equipment |
| 09.2 Other major durables for recreation and culture |
| 09.3 Other recreational items, gardens and pets |
| 09.4 Recreational and cultural services |
| 09.5 Books, newspapers and stationery |
| 09.6 Package holidays |
| 10.0 Education |
| 11.1 Catering services |
| 11.2 Accommodation services |
| 12.1 Personal care |
| 12.3 Personal effects nec |
| 12.4 Social protection |
| 12.5 Insurance |
| 12.6 Financial services nec |
| 12.7 Other services nec |

**APPENDIX 3**

ONS alcohol price item descriptions and average price were used to determine whether the item was sold on- or off-trade.

| **Item ID** | **Item Description** | **On-trade** | **2007** | | **2008** | | **2009** | | **2010** | |
| --- | --- | --- | --- | --- | --- | --- | --- | --- | --- | --- |
|  |  |  | **Mean** | **SD** | **Mean** | **SD** | **Mean** | **SD** | **Mean** | **SD** |
| 310218 | APPLE CIDER 500-750ML 4.5-5.5% | No |  |  |  |  |  |  | £1.83 | 0.12 |
| 310207 | BITTER-4CANS-440-500ML | No | £3.92 | 0.17 | £3.88 | 0.18 | £3.85 | 0.20 | £3.86 | 0.18 |
| 310314 | BOTTLE OF CHAMPAGNE | No | £32.07 | 3.48 | £32.54 | 3.29 | £33.86 | 3.76 | £34.82 | 3.78 |
| 310423 | BOTTLE OF CHAMPAGNE 75 CL | No | £21.92 | 1.28 | £22.53 | 1.34 | £24.42 | 1.48 | £25.89 | 1.55 |
| 310112 | BOTTLE OF LAGER IN NIGHTCLUB | Yes | £2.93 | 0.26 | £2.99 | 0.28 | £3.09 | 0.32 | £3.20 | 0.34 |
| 310315 | BOTTLE OF WINE 70-75CL | Yes | £11.80 | 0.86 | £12.29 | 0.57 | £12.61 | 0.46 | £12.96 | 0.58 |
| 310111 | BOTTLED PREMIUM LAGER 4.3-7.5% | Yes | £2.43 | 0.12 | £2.50 | 0.13 | £2.60 | 0.14 | £2.69 | 0.15 |
| 310405 | BRANDY 70CL BOTTLE | No | £13.27 | 1.11 | £13.38 | 1.15 | £14.31 | 1.15 | £15.03 | 1.39 |
| 310205 | CIDER 1-2 LITRE BOTTLE | No | £2.47 | 0.23 | £2.63 | 0.22 | £2.71 | 0.17 |  |  |
| 310106 | CIDER-1/2PT OR 275-340ML BOT | Yes | £1.30 | 0.07 | £1.33 | 0.08 | £1.38 | 0.08 |  |  |
| 310113 | CIDER-PER PINT OR 500-568ML | Yes |  |  |  |  |  |  | £2.85 | 0.14 |
| 310102 | DRAUGHT BITTER (PER PINT) | Yes | £2.18 | 0.14 | £2.26 | 0.14 | £2.35 | 0.16 | £2.44 | 0.16 |
| 310104 | DRAUGHT STOUT PER PINT | Yes | £2.55 | 0.14 | £2.63 | 0.14 | £2.75 | 0.15 | £2.87 | 0.14 |
| 310406 | FORTIFIED WINE (70-75CL) | No | £5.69 | 0.60 | £5.85 | 0.60 | £6.36 | 0.80 | £6.60 | 0.67 |
| 310109 | LAGER - PINT 3.4-4.2% | Yes | £2.34 | 0.12 | £2.42 | 0.13 | £2.53 | 0.14 | £2.63 | 0.13 |
| 310216 | LAGER 12 - 24 CANS (440-500ML) | No | £7.85 | 0.22 | £8.06 | 0.34 | £8.44 | 0.42 | £9.22 | 0.33 |
| 310217 | LAGER 12-24 BOTTLE (250-300ML) | No |  |  |  |  | £11.23 | 1.32 | £11.51 | 1.55 |
| 310215 | LAGER 4 BOTTLES- PREMIUM | No | £3.80 | 0.17 | £3.90 | 0.17 | £3.91 | 0.21 | £4.00 | 0.14 |
| 310213 | LAGER 4 CANS- PREMIUM 4.3-7.5% | No | £4.11 | 0.14 | £4.10 | 0.13 | £4.12 | 0.18 | £4.35 | 0.17 |
| 310214 | LAGER STUBBIES 4.3-7.5% ABV | Yes | £10.26 | 0.96 | £10.08 | 1.25 |  |  |  |  |
| 310307 | LIQUEUR PER NIP SPECIFY ML | Yes | £1.93 | 0.15 | £1.99 | 0.16 | £2.07 | 0.14 | £2.15 | 0.15 |
| 310110 | PREMIUM LAGER - PINT 4.3-7.5% | Yes | £2.61 | 0.12 | £2.70 | 0.13 | £2.82 | 0.14 | £2.94 | 0.14 |
| 310421 | RED WINE- EUROPEAN 75CL | No | £4.73 | 0.33 | £4.63 | 0.38 | £4.75 | 0.39 | £5.16 | 0.38 |
| 310422 | RED WINE- NEW WORLD 75CL | No | £4.82 | 0.24 | £4.94 | 0.25 | £5.27 | 0.32 | £5.64 | 0.32 |
| 310425 | ROSE WINE-75CL BOTTLE | No |  |  |  |  |  |  | £5.57 | 0.74 |
| 310417 | SPIRIT BASED DRINK 250-330MLS | Yes | £1.32 | 0.08 | £1.26 | 0.08 | £1.24 | 0.09 | £1.24 | 0.08 |
| 310309 | SPIRIT BASED DRINK 275ML | Yes | £2.58 | 0.15 | £2.65 | 0.12 | £2.73 | 0.13 | £2.80 | 0.14 |
| 310302 | VODKA (PER NIP) SPECIFY ML | Yes | £1.80 | 0.15 | £1.87 | 0.15 | £1.99 | 0.15 | £2.08 | 0.16 |
| 310403 | VODKA-70 CL BOTTLE | No | £9.99 | 0.31 | £10.22 | 0.34 | £10.53 | 0.55 | £10.88 | 0.51 |
| 310301 | WHISKY (PER NIP) SPECIFY ML | Yes | £1.81 | 0.15 | £1.88 | 0.16 | £1.96 | 0.15 | £2.03 | 0.15 |
| 310401 | WHISKY-70 CL BOTTLE | No | £11.82 | 0.49 | £12.10 | 0.58 | £13.02 | 0.65 | £13.36 | 0.57 |
| 310419 | WHITE WINE- EUROPEAN 75CL | No | £4.26 | 0.31 | £4.22 | 0.29 | £4.64 | 0.41 | £4.99 | 0.41 |
| 310420 | WHITE WINE- NEW WORLD 75CL | No | £5.37 | 0.18 | £5.53 | 0.19 | £5.67 | 0.26 | £5.87 | 0.27 |
| 310310 | WINE, PER 175 - 250 ML SERVING | Yes | £2.44 | 0.14 | £2.52 | 0.14 | £2.56 | 0.25 | £2.66 | 0.31 |

**APPENDIX 4**

Alcohol-related ICD 10 Codes

This is a summary of the distinct primary/main diagnosis codes a participant has had recorded across all their hospital inpatient records. Diagnoses are coded according to the International Classification of Diseases version-10 (ICD-10) and includes primary, secondary and external causes.

| Code | Description |
| --- | --- |
| E24.4 | Alcohol-induced pseudo-Cushing syndrome |
| E51.2 | Wernicke encephalopathy |
| F10.0 | Mental and behavioural disorders due to use of alcohol: Acute intoxication |
| F10.1 | Mental and behavioural disorders due to use of alcohol: Harmful use |
| F10.2 | Mental and behavioural disorders due to use of alcohol: Dependence syndrome |
| F10.3 | Mental and behavioural disorders due to use of alcohol: Withdrawal state |
| F10.4 | Mental and behavioural disorders due to use of alcohol: Withdrawal state with delirium |
| F10.5 | Mental and behavioural disorders due to use of alcohol: Psychotic disorder |
| F10.6 | Mental and behavioural disorders due to use of alcohol: Amnesic syndrome |
| F10.7 | Mental and behavioural disorders due to use of alcohol: Residual and late-onset psychotic disorder |
| F10.8 | Mental and behavioural disorders due to use of alcohol: Other mental and behavioural disorders |
| F10.9 | Mental and behavioural disorders due to use of alcohol: Unspecified mental and behavioural disorder |
| G31.2 | Degeneration of nervous system due to alcohol |
| G40.5 | Special epileptic syndromes |
| G62.1 | Alcoholic polyneuropathy |
| G72.1 | Alcoholic myopathy |
| I42.6 | Alcoholic cardiomyopathy |
| K29.2 | Alcoholic gastritis |
| K70.0 | Alcoholic fatty liver |
| K70.1 | Alcoholic hepatitis |
| K70.2 | Alcoholic fibrosis and sclerosis of liver |
| K70.3 | Alcoholic cirrhosis of liver |
| K70.4 | Alcoholic hepatic failure |
| K70.9 | Alcoholic liver disease, unspecified |
| K85.2 | Alcohol-induced acute pancreatitis |
| K86.0 | Alcohol-induced chronic pancreatitis |
| O35.4 | Maternal care for (suspected) damage to fetus from alcohol |
| R78.0 | Finding of alcohol in blood |
| T51.0 | Toxic effect: Ethanol |
| X45.0 | Accidental poisoning by and exposure to alcohol home while engaged in sports activity |
| X45.1 | Accidental poisoning by and exposure to alcohol home while engaged in leisure activity |
| X45.2 | Accidental poisoning by and exposure to alcohol home while working for income |
| X45.3 | Accidental poisoning by and exposure to alcohol home while engaged in other types of work |
| X45.4 | Accidental poisoning by and exposure to alcohol home while resting sleeping eating or engaging in other |
| X45.8 | Accidental poisoning by and exposure to alcohol home while engaged in other specified activities |
| X45.9 | Accidental poisoning by and exposure to alcohol home during unspecified activity |
| X65.0 | Intentional self-poisoning by and exposure to alcohol home while engaged in sports activity |
| X65.1 | Intentional self-poisoning by and exposure to alcohol home while engaged in leisure activity |
| X65.2 | Intentional self-poisoning by and exposure to alcohol home while working for income |
| X65.4 | Intentional self-poisoning by and exposure to alcohol home while resting sleeping eating or engaging |
| X65.8 | Intentional self-poisoning by and exposure to alcohol home while engaged in other specified activities |
| X65.9 | Intentional self-poisoning by and exposure to alcohol home during unspecified activity |
| Y15.0 | Poisoning by and exposure to alcohol, undetermined intent, home |
| Y15.1 | Poisoning by and exposure to alcohol, undetermined intent, residential institution |
| Y15.2 | Poisoning by and exposure to alcohol, undetermined intent, school, other institution |
| Y15.3 | Poisoning by and exposure to alcohol, undetermined intent, sports and athletics area |
| Y15.4 | Poisoning by and exposure to alcohol, undetermined intent, street and highway |
| Y15.5 | Poisoning by and exposure to alcohol, undetermined intent, trade and service area |
| Y15.6 | Poisoning by and exposure to alcohol, undetermined intent, industrial and construction area |
| Y15.7 | Poisoning by and exposure to alcohol, undetermined intent, farm |
| Y15.8 | Poisoning by and exposure to alcohol, undetermined intent, other specified places |
| Y15.9 | Poisoning by and exposure to alcohol, undetermined intent, unspecified place |
| Y90.0 | Blood alcohol level of less than 20 mg/100 ml |
| Y90.1 | Blood alcohol level of 20-39 mg/100 ml |
| Y90.2 | Blood alcohol level of 40-59 mg/100 ml |
| Y90.3 | Blood alcohol level of 60-79 mg/100 ml |
| Y90.4 | Blood alcohol level of 80-99 mg/100 ml |
| Y90.5 | Blood alcohol level of 100-119 mg/100 ml |
| Y90.6 | Blood alcohol level of 120-199 mg/100 ml |
| Y90.7 | Blood alcohol level of 200-239 mg/100 ml |
| Y90.8 | Blood alcohol level of 240 mg/100 ml or more |
| Y90.9 | Presence of alcohol in blood, level not specified |
| Y91.0 | Mild alcohol intoxication |
| Y91.1 | Moderate alcohol intoxication |
| Y91.2 | Severe alcohol intoxication |
| Y91.3 | Very severe alcohol intoxication |
| Y91.9 | Alcohol involvement, not otherwise specified |
| Z50.2 | Alcohol rehabilitation |
| Z71.4 | Alcohol abuse counselling and surveillance |
| Z72.1 | Alcohol use |
